# Research on Cantilever Beam Roller Tension Sensor Based on Surface Acoustic Wave

**DOI:** 10.3390/mi16091044

**Published:** 2025-09-11

**Authors:** Yang Feng, Bingkun Zhang, Yang Chen, Ben Wang, Hua Xia, Haoda Yu, Xulehan Yu, Pengfei Yang

**Affiliations:** 1 School of Information Science and Engineering (SISE), Hangzhou Normal University, Hangzhou 311121, China; zhangbingkun08@stu.hznu.edu.cn (B.Z.); 20170056@hznu.edu.cn (B.W.); xiahua@stu.hznu.edu.cn (H.X.); yuhaoda0205@stu.hznu.edu.cn (H.Y.); 2024210214052@stu.hznu.edu.cn (X.Y.); 2 Mobile Health Management System Engineering Research Center of the Ministry of Education, Hangzhou 311121, China; 3 Zhejiang-Cyprus Smart City and Mobile Health Joint Laboratory, Hangzhou 311121, China; cheny@zhundao.net; 4 Hangzhou Zhundao Information Technology Co., Ltd., Hangzhou 311121, China

**Keywords:** tension sensors, surface acoustic wave, cantilever beam, finite element method, GA-PSO-BP, temperature compensation

## Abstract

This paper presents a design method for a continuous tension detection sensor based on a cantilever beam structure and compensates for the temperature drift of a SAW sensor based on a neural network algorithm. Firstly, a novel cantilever beam roller structure is proposed to significantly enhance the sensitivity of the transmission of silk thread tension to a SAW tension sensor. Secondly, to improve the sensitivity of the SAW tension sensor, the COMSOL finite element method (FEM) is employed for simulation to determine the optimal IDT placement. An unbalanced split IDT design is utilized to suppress potential parasitic responses. Finally, the designed sensor is tested, and a GA-PSO-BP algorithm is employed to fit the test data for temperature compensation. The experimental results demonstrate that the temperature sensitivity coefficient of the data optimized by the GA-PSO-BP algorithm is reduced by an order of magnitude compared to the raw data, with reductions of $6.0409\times 10^{-3}\;{{}^{\circ}C}^{-1}$ and $3.0312\times 10^{-3}{\;{}^{\circ}C}^{-1}$ compared to the BP neural network and the PSO-BP algorithm, respectively. The average output error of the optimized data is reduced by 5.748% compared to the sensor measurement data, and it is also lower than both the BP neural network and the PSO-BP algorithm. It provides new design ideas for the development of tension sensors.

## 1. Introduction

Silk, a historically significant and culturally valuable textile, is highly prized in clothing and decoration for its unique luster, softness, and breathability [1]. Tension control during silk production is critical for maintaining product quality. Each stage, from reeling to twisting to weaving, demands precise tension monitoring and adjustment. Insufficient tension can cause slackness and wrinkling, while excessive tension may lead to breakage, both of which negatively impact production efficiency and final product quality [2,3].

In recent years, the rapid development of Surface Acoustic Wave (SAW) sensor technology has offered new solutions for tension measurement in silk production. SAW sensors detect changes in physical quantities through acoustic wave propagation on the surface of piezoelectric materials. They feature high sensitivity, wireless operation, and strong anti-electromagnetic interference capability [4,5]. SAW tension sensors can convert strain signals into frequency changes for contactless tension measurement. This approach prevents damage to silk fibers from traditional contact sensors and enhances measurement accuracy and stability.

However, a key challenge in using SAW sensors for silk tension measurement is temperature compensation. The piezoelectric materials used in SAW sensors are sensitive to temperature changes, which can alter the output frequency of the sensor and thus affect measurement precision [6,7]. Temperature fluctuations in silk production workshops, caused by seasonal changes or equipment operation, necessitate effective temperature compensation strategies to ensure the accuracy and reliability of SAW tension sensors.

To address the issue of temperature compensation, Lu, Guo, and U. Youbi et al. [7,8,9] employed a hardware compensation scheme. Although this method can suppress temperature interference to some extent, it has limitations such as a narrow applicable range, high cost, and complex debugging requirements. In contrast, software compensation methods offer advantages such as low cost, high flexibility, reliability, and precision. Sun et al. [10] proposed a temperature compensation model for silicon piezoresistive pressure sensors based on a particle swarm optimization (PSO) algorithm in combination with a BP neural network. Feng et al. [11] proposed a PSO-LSSVM algorithm to compensate for temperature errors in SAW tension sensors.

To satisfy the tension control requirements of silk production, this study focuses on developing a cantilever beam roller SAW tension sensor. It also proposes using a GA-PSO-BP algorithm to compensate for the temperature, which induced errors in SAW tension sensors. By optimizing the sensor’s design and structure, the goal is to enhance the measurement accuracy and stability of silk tension. Moreover, by designing a proper temperature compensation strategy that considers real-world production temperature variations, the sensor can ensure measurement accuracy across different temperatures. Experimental results, compared with those of traditional BP neural networks and PSO-BP algorithms, show that the GA-PSO-BP algorithm offers superior prediction performance. This research not only enhances the tension detection accuracy of textile silk products but also provides a new design concept of cantilever beam structure for high-precision tension detection equipment.

The structure of this paper is as follows: after the introduction in Section 1, Section 2 covers the principles of cantilever beam roller SAW tension sensors and temperature compensation algorithms. Section 3 presents the design and simulation of the cantilever beam roller SAW tension sensor. In Section 4, the data measurement of the cantilever beam roller SAW tension sensor is completed, and analysis of temperature compensation is provided. The conclusion of this paper can be found in Section 5.

## 2. Principles of SAW Tension Sensor and GA-PSO-BP Algorithm

### 2.1. Principle of the Cantilever Beam Roller SAW Structure

The piezoelectric materials (e.g., quartz crystals, lithium niobate) of SAW sensors are subjected to mechanical stress, and they generate electric charges. Conversely, applying an electric field can cause mechanical deformation [12]. In SAW sensors, interdigital transducers (IDTs) are used to excite and detect SAWs. Upon receiving an electromagnetic signal, the IDT, leveraging the piezoelectric effect, converts electrical signals into acoustic surface waves, which then propagate along the surface of the piezoelectric material.

**Figure 1:**
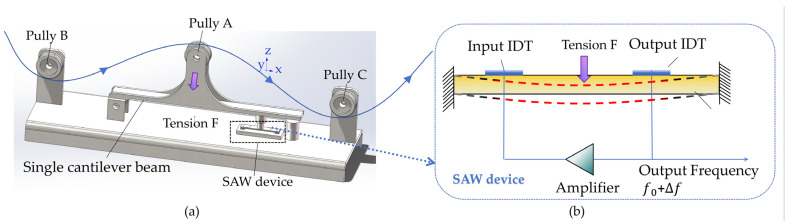
(**a**) Suspended beam structural diagram. (**b**) Schematic diagram of SAW device structure.

The schematic diagram of the cantilever beam roller SAW tension sensor structure proposed in this paper is shown in Figure 1a. The cantilever beam roller SAW tension sensor comprises three guide rollers: roller A, roller B, and roller C. The resultant tension F_R_ of the yarn equals the yarn tension F. Roller A is fixed to the cantilever beam, and the tension induces deformation in the beam, transmitting the force to the SAW device. As shown in Figure 1b, the SAW device consists of a doubly supported piezoelectric substrate, an input IDT, and an output IDT. The SAW oscillator is formed by connecting the SAW device to an amplifier.

**Figure 2:**
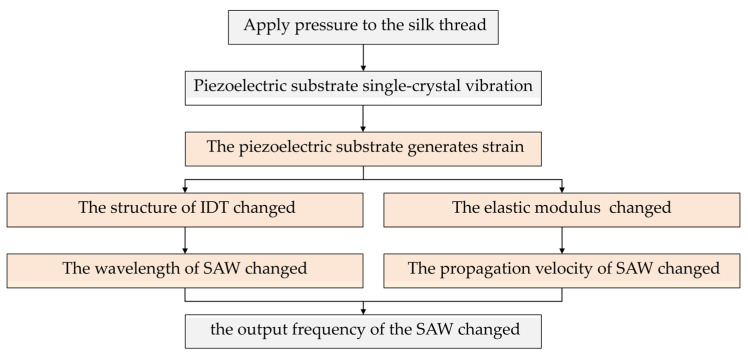
SAW tension sensor working flow chart.

When the yarn tension F applies on the piezoelectric substrate of the single-cantilever-beam SAW sensor through the guide rollers and rods, the geometry of the input and output IDTs (finger width and finger spacing) and the elastic modulus of the piezoelectric substrate are altered. These changes induce a variation in the output frequency of the SAW oscillator (Figure 2). In summary, the frequency shift of the SAW oscillator is linearly proportional to the yarn tension F, thereby enabling accurate yarn tension measurement [13].

The output frequency of the SAW oscillator can be expressed by

(1) $$f=f_{0}+{\Delta f}_{F}\;$$

where $f_{0}$ is the oscillator’s output frequency when the applied tension F=0, and ${\Delta f}_{F}$ is the frequency shift induced when F≠0. This frequency shift ${\Delta f}_{F}$ is linearly related to the tension *F*:

(2) $${\Delta f}_{F}=\alpha F$$

in which *α* is a constant. Alternatively, the frequency shift can be written as

(3) $$\Delta f_{F}=f-f_{0}=f_{0}\frac{1+k^{\prime }\varepsilon }{1+\varepsilon }-f_{0}=f_{0}\frac{\varepsilon (k^{\prime }-1)}{1+\varepsilon }$$

For the SAW oscillator, the center frequency $f_{0}$ and the elastic-stiffness constant $k^{\prime }$ of the piezoelectric substrate are known. Therefore, the frequency shift $\Delta f_{F}$ is determined solely by the strain ε in the piezoelectric material.

### 2.2. Principle of GA-PSO-BP Algorithm

#### 2.2.1. BP Neural Network

A BP neural network is a multi-layer feedforward neural network that optimizes weights and thresholds through the backpropagation algorithm. Its structure includes an input layer, a hidden layer, and an output layer [14]. Leveraging this advantage, the BP neural network can address complex nonlinear issues such as temperature compensation for sensors, thereby enhancing the measurement accuracy and reliability of the sensors [15].

Forward propagation is the process by which input data are passed through each layer of the network and computed to produce an output. The forward-propagation formulas are as follows:

(4) $$\left\{\begin{array}{c} z_{j}^{l}=\sum_{i=1}^{n_{l-1}}w_{ji}^{l}a_{i}^{l-1}+b_{j}^{l}\\ a_{j}^{l}=f\left(z_{j}^{l}\right) \end{array}\right.$$

where the superscripts denote the layer index and the subscripts denote the neuron index.

The essence of backpropagation is to quantify the discrepancy between the network’s output and the ground-truth target, propagate this error backward, and adjust every parameter (weights and biases) so as to minimize the overall loss.

For the *m*-th hidden layer, the error can be calculated using the backpropagation formula:

(5) $$\delta_{j}^{m}=\left(\sum_{k=1}^{n_{m+1}}\delta_{k}^{m+1}w_{kj}^{m+1}\right)\cdot \sigma^{\prime }\left(z_{j}^{m}\right)$$

For the output layer of the L-th layer, the error is

(6) $$\delta_{j}^{L}=\frac{\partial J}{\partial z_{j}^{L}}=y_{j1}-y_{j}$$

The gradient expressions of weights and biases are

(7) $$\left\{\begin{array}{c} \frac{\partial J}{\partial w_{ji}^{l}}=a_{i}^{l-1}\delta_{j}^{l}\\ \frac{\partial J}{\partial b_{j}^{l}}=\delta_{j}^{l} \end{array}\right.$$

Using gradient descent, the parameters are updated as

(8) $$\left\{\begin{array}{c} w_{ji}^{l}\leftarrow w_{ji}^{l}-\eta a_{i}^{l-1}d_{j}^{l}\\ b_{j}^{l}\leftarrow b_{j}^{l}-\eta d_{j}^{l} \end{array}\right.$$

The relevant parameters are given in Table 1. The core of the BP neural network lies in performing forward propagation to calculate the output of the network, using the cost function to quantify the error, calculating the gradient through backpropagation, and then updating the weights and biases to minimize this error. Through repeated iterations, the network’s performance is progressively optimized. During the training of BP neural networks, the error surface frequently contains multiple local minima. When the network training process falls into these local minima, further weight adjustments barely reduce the error and may even cause it to stall. This prevents the network from reaching the global optimum, thereby undermining its generalization and performance. To address the BP neural network’s shortcomings of being trapped in local minima and having slow convergence, it is essential to develop optimization strategies. These strategies should enable the network to escape local minima, enhance overall performance, and significantly accelerate convergence.

#### 2.2.2. PSO-BP Algorithm

At the end of the last century, J. Kennedy et al. [16] proposed the particle swarm optimization (PSO) algorithm, a meta-heuristic approach for solving optimization problems. Inspired by the modeling and simulation of bird-flocking behavior, PSO-based network optimization can expand the search scope and prevent BP neural networks from falling into local optima [17].

When analyzing the principles of the particle-swarm algorithm, the following basic assumptions are made: in a *D* dimensional space, there exists a collection of N particles. This collection exchanges and updates information through specific rules to achieve optimization. Dimension *D* is determined as

(9) D=l×m+m×n+m+n

In this space, the position $X_{i}$ and velocity $V_{i}$ of the i-th particle are expressed as

(10) $$\left\{\begin{array}{cc} X_{i}=\left(x_{i1},x_{i2},\ldots ,x_{iD}\right), & i\in [1,2,\ldots ,N]\\ V_{i}=\left(v_{i1},v_{i2},\ldots ,v_{iD}\right), & i\in [1,2,\ldots ,N] \end{array}\right.$$

To avoid a blind search in the space, $X_{i}$ and $V_{i}$ are limited by maximum position $X_{max}$ and maximum velocity $V_{max}$. $X_{i}$ ranges within ${[-X}_{max},X_{max}]$, and $V_{i}$ ranges within ${[-V}_{max},V_{max}]$.

In each iteration, a neural network predicts the particle’s position to calculate fitness. Comparing fitness values updates each particle’s local optimum and the swarm’s global optimum. All particles search independently until the maximum iterations are reached, and the individual extremum $P_{i}$ is recorded:

(11) $$P_{i}=\left(p_{i1},p_{i2},\ldots ,p_{iD}\right),\;i\in [1,2,\ldots ,N]$$

During the particle-swarm operation, each particle interacts and shares information. The best global position found by all particles is called the global extremum $G_{g}$:

(12) $$G_{g}=\left(G_{g1},G_{g2},\ldots ,G_{gD}\right),i\in [1,2,\ldots ,N]$$

By analyzing $P_{i}$ and $G_{g}$, particles update their velocity and position as follows:

(13) $$\left\{\begin{array}{c} v_{i+1}=\omega v_{i}+c_{1}r_{1}\left(P_{i}-x_{i}\right)+c_{2}r_{2}\left(G_{g}-x_{i}\right)\\ x_{i+1}=x_{i}+v_{i+1} \end{array}\right.$$

The relevant parameters are given in Table 2. With its unique velocity-guidance mechanism, PSO enhances search speed and demonstrates strong memory. Optimal particles record and share their position information. However, the PSO-BP algorithm may have low precision due to the lack of real-time dynamic velocity adjustment and sensitivity to parameter settings.

#### 2.2.3. GA-PSO-BP Algorithm

To enhance optimization, the GA-PSO integrated algorithm combines the genetic mechanism of genetic algorithms with the particle swarm algorithm.

Based on the PSO algorithm, each particle’s velocity and position are updated by considering the particle’s historical best position and the swarm’s best position, following the standard PSO update rules.

Drawing from the GA algorithm, some individuals are selected as parents, and new offspring are generated through crossover and mutation. An elite strategy is employed, selecting particles with the smallest fitness values from the global and historical optimal solutions as parents in the genetic offspring.

The offspring are merged with the current population, fitness values are recalculated, and particles with the smallest fitness values are chosen as the new generation. When the termination condition is met, the global optimal solution is outputted.

The GA-PSO algorithm integrates the global search of GA and the local search of PSO, effectively solving complex optimization problems while maintaining excellent search performance and convergence.

The training of a GA-PSO-BP neural network involves the following steps:

(1) Initialization: Create a BP neural network. Initialize the population (for GA), where each individual represents the network’s weights and biases. Initialize the particle swarm (for PSO), with each particle also representing the weights and biases.
(2) GA optimization: Generate new individuals by applying selection, crossover, and mutation operations to the network’s weights and biases. Evaluate each individual’s fitness using the network’s error or loss function.
(3) PSO adjustment: Initialize or update the particle swarm’s positions based on the GA-processed population. Update each particle’s velocity and position using historical best positions (individual and global). Update the network’s weights and biases accordingly.
(4) BP training: Train the BP neural network using the updated weights and biases, adjusting them through backpropagation based on the loss function.
(5) Iteration check: Repeat steps (2)–(4) until a stopping condition is met, such as a maximum number of iterations or a minimum error threshold.

The result is a neural network with enhanced learning and generalization capabilities, less prone to local optima.

## 3. Design and Simulation of SAW Tension Sensor

### 3.1. Design of SAW Device

**Figure 3:**
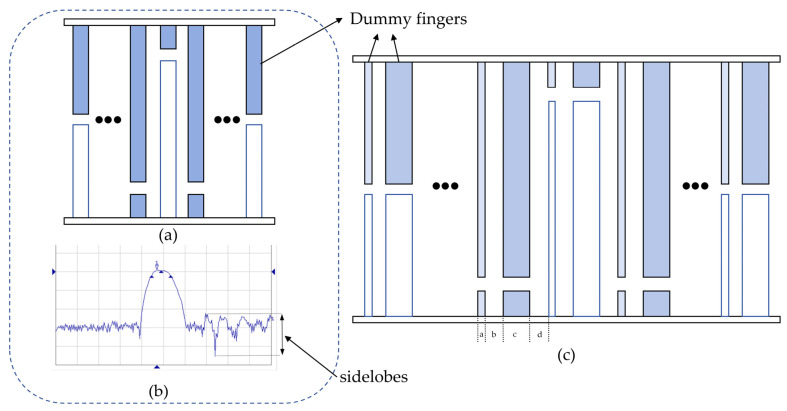
(**a**) Single-electrode IDTs. (**b**) Diagram showing excessive sidelobes in the frequency of single-electrode IDTs. (**c**) Unbalanced split-electrode IDTs.

Single-electrode IDTs are shown in Figure 3a [18], which is very likely to cause a second-order effect, causing edge reflections and increasing the sidelobe levels of the SAW sensor. Excessive sidelobes in the frequency of single-electrode IDTs are shown in Figure 3b. To mitigate sidelobes, an unbalanced split design is proposed, as shown in Figure 3c.

In Figure 3c, *a* and *c* represent the widths of the segmented electrodes, while *b* and *d* represent the electrode spacings. These parameters are related to the wavelength λ as follows:

(14) $$a=\frac{1}{16}\lambda ,\;b=\frac{2}{16}\lambda ,\;c=\frac{3}{16}\lambda ,\;d=\frac{2}{16}\lambda$$

To further suppress side lobes, the input IDT’s electrode overlap envelope is weighted according to the Hamming [19,20] function:

(15) $$\begin{array}{cc} h\left(t\right) & =0.08+\left(1-0.08\right)\cos^{2}\frac{\pi t}{2\tau }\\ & =0.54+0.46\cos\frac{\pi t}{\tau },\;\tau \leq t \end{array}$$

The output transducer adopts a uniform split design, as shown in Figure 3a. Given ${\;N}_{out}>20$, the number of output IDT electrode pairs is chosen as $N_{out}=24$.

The input and output IDTs can be defined as

(16) $$\left\{\begin{array}{c} H_{in}(f)=\sum_{n=1}^{N_{i}}W\left(0.54+0.46\cos\frac{\omega x_{n}/v}{2.1}\right)e^{-jfx_{n}/v}\\ H_{out}(f)=\sum_{m=1}^{N_{0}}We^{-jfx_{m}/v} \end{array}\right.$$

The overall response H(f) is the sum of the input and output IDT responses:

(17) $$H(f)=H_{out}(f)H_{in}(f)$$

The −3 dB bandwidth is given by

(18) $$BW=\frac{\Delta f_{-3\mathrm{dB}}}{f_{0}}=\frac{\sqrt{2}}{2}|H(f)|=\frac{\sqrt{2}}{2}|H_{in}(f)||H_{out}(f)|$$

With ${\;N}_{out}=24$, the relationship between BW and ${\;N}_{in}$ is derived as

(19) $$BW=\frac{\Delta f_{-3\mathrm{dB}}}{f_{0}}=\frac{1.2288}{60}=\frac{1.2036}{N_{in}}$$

For a designed SAW tension sensor bandwidth of BW=2.04%, the number of input IDT pairs is determined to be ${\;N}_{in}=59$.

The parameters related to the formulas are shown in Table 3.

The design parameters of the SAW tension sensor based on the cantilever beam structure are shown in Table 4.

### 3.2. FEM of SAW Device

To achieve enhanced sensitivity of the SAW tension sensor and accurately simulate the sensor’s behavior under complex conditions, this paper first conducts simulation analysis of the sensor. The finite element method (FEM) employed in this paper is an innovative approach to solving elastic–plastic problems. The fundamental concept of the FEM [21] involves discretizing a continuous geometry into finite elements, applying boundary conditions, and solving the equations to obtain numerical solutions, thereby transforming an infinite degree-of-freedom problem into a finite degree-of-freedom problem.

COMSOL Multiphysics 6.2 software is used to model a cantilever beam SAW tension sensor and determine the strain distribution in the piezoelectric substrate. In order to make the experimental results more accurate, the size of the grid cells was set to be “extremely fine” (1110 thousand domain units). ST-X quartz crystal is chosen as the piezoelectric material because it has superior strain performance compared to other crystals [22,23]. The materials and design parameters of the SAW tension sensor are summarized in Table 5.

**Figure 4:**
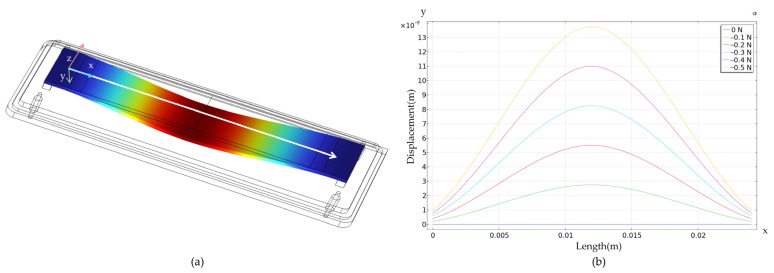

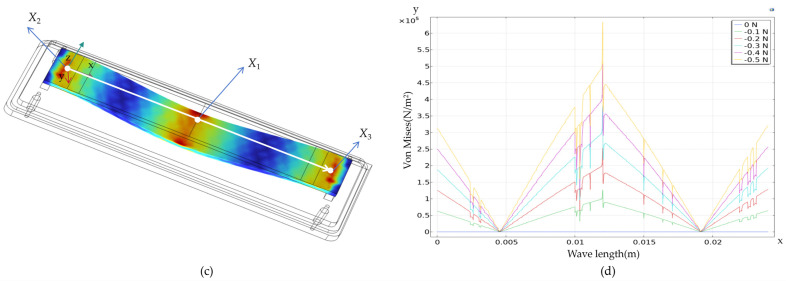
(**a**) Displacement diagram of the SAW tension sensor (F = 0.5 N). (**b**) Displacement curve of the SAW tension sensor. (**c**) Strain diagram of the SAW tension sensor (F = 0.5 N). (**d**) Strain curve of the SAW tension sensor.

After setting up the model parameters, meshing, and conastraints, a tensile force of F = 0.5 N is applied to the cantilever beam. The tensile force F = 0.5 N was selected as it represents the upper limit of typical silk thread tension during weaving processes (0.3–0.6 N) [1]. This value ensures validation of sensor performance under high-stress conditions while operating within the linear range of the sensor (0–1 N). The force F is transmitted to the cantilever beam through the cylindrical surface of the yarn guide roller A, with the contact surface being a line contact. The resulting deformation and stress distribution are shown in Figure 4a,c, where blue indicates the minimum strain and red the maximum strain regions. Figure 4b shows the displacement variation from 0 to 0.5 N at the center line of the base. This data was obtained through parameter scanning. Figure 4d shows corresponding stress changes in the substrate under the same force range.

The FEM of the piezoelectric substrate shows that strain and displacement peak at the center of the piezoelectric plate, specifically at point X_1_ in Figure 4c. Due to the excessively high curvature, the IDTs may become asymmetric in shape [24]. Therefore, the input and output IDTs are placed at points X2 and X3, respectively. These points exhibit significant strain and minimal deformation.

**Figure 5:**
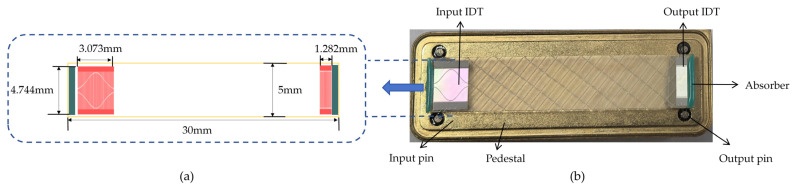
(**a**) Design of the piezoelectric substrate’s size. (**b**) SAW device fabricated on the ST-X quartz substrate.

As shown in Figure 5a, the appropriate size of the SAW transducer was designed, and the L-edit 16.3 software was used to draw the mask layout diagram of the SAW transducer. Figure 5b is the physical diagram of the SAW tension sensor.

## 4. Data Measurement and Processing

### 4.1. Data Measurement

**Figure 6:**
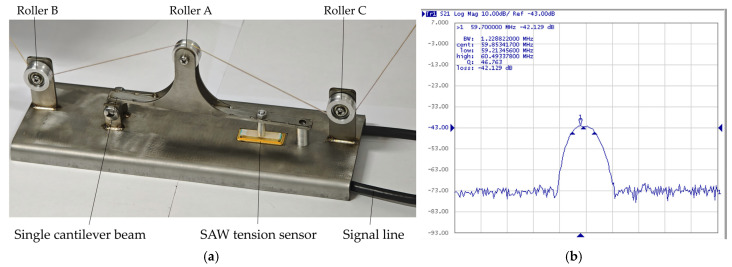
(**a**) The construction diagram of the cantilever beam roller SAW yarn tension sensor measurement system. (**b**) Frequency response characteristics of the SAW sensor.

Based on the characteristics of the cantilever beam roller SAW tension sensor and the feasibility of the simulation results, we designed and developed a measurement system to explore the relationship between input variables and output variables, as shown in Figure 6a. In Figure 6b, Keysight E5061A (Keysight Technologies, America) was used to measure the frequency response characteristics of the SAW sensor.

To characterize the sensor’s output under varying tensions, this paper applied tensions ranging from 0 to 1 N in 0.1 N increments and tested the SAW sensor at different temperatures to assess thermal effects. High-precision temperature sensors were utilized to set and stabilize the room temperature of the entire testing environment. Some test data for the SAW winding tension sensor at various temperatures are listed in Table 6.

The temperature sensitivity coefficient is utilized to determine whether the temperature has any impact on the accuracy of the SAW sensor. The temperature sensitivity coefficient, $\alpha_{s}$, defined as

(20) $$\alpha_{s}=\frac{\Delta d_{max}}{\Delta T\cdot \Delta f(FS)}$$

quantifies the temperature impact on the sensor’s accuracy. Here, $\;\Delta d_{max}$ is the full-range winding tension across temperatures, ΔT is the maximum temperature change, and Δf is the frequency shift range. A larger $\alpha_{s}$ indicates a greater thermal influence on the sensor’s precision. From the table data, this paper selected F = 1.0 N, $\Delta d_{max}=14678-11227=3551\;Hz$, ΔT=40−20 °C, so

(21) $$\alpha_{s}=\frac{3551}{20\times 14678}=1.2096\times 10^{-2}\;{℃}^{-1}$$

The results show that temperature variations significantly affect the SAW tension sensor, so temperature compensation is essential.

### 4.2. Data Processing

**Figure 7:**
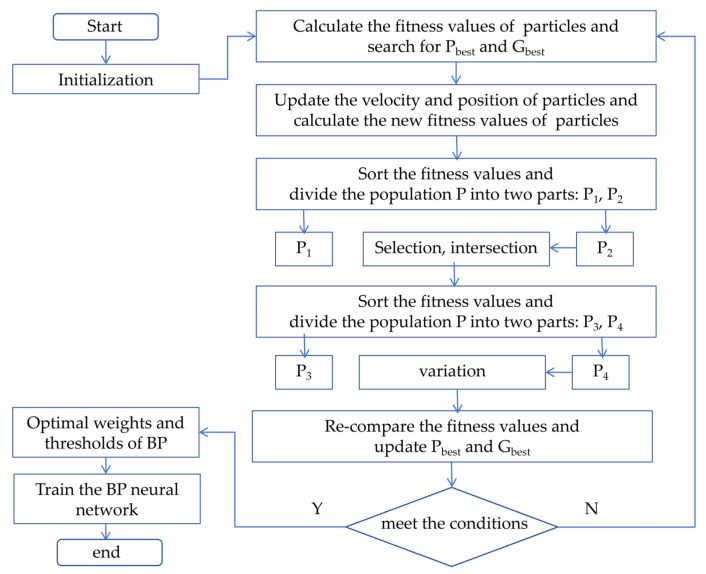
The implementation steps of the GA-PSO-BP algorithm.

A GA-PSO-BP algorithm is proposed for data processing to mitigate the thermal impact on the SAW tension sensor and reduce measurement errors. By combining the global search of GA with the local search of PSO, it effectively addresses complex, high-dimensional, nonlinear optimization problems while ensuring excellent convergence and search performance. The implementation steps of the GA-PSO-BP algorithm are illustrated in Figure 7.

Table 7 shows the data after GA-PSO-BP algorithm temperature compensation. From the data in Table 7, the compensated temperature sensitivity coefficient $\alpha_{s}$ was calculated. Taking the data at F = 1.0 N as an example, $\Delta d_{max}=12323-12003=320\;Hz$, and ΔT=40−20 °C, so

(22) $$\alpha_{s1}=\frac{320}{20\times 12323}=1.2984\times 10^{-3}\;{℃}^{-1}$$

### 4.3. Data Comparison

To facilitate data comparison, this paper conducted a comparative analysis on the same dataset, comparing the traditional measurement values, BP neural network, PSO-BP algorithm, and GA-PSO-BP algorithm. All training and testing samples were collected from the SAW sensor’s measured frequency response data under different temperatures (20–40 °C) and different tension levels (0–1.0 N), as shown in Table 6. An 80–20% random split was used for training and testing sets, ensuring all algorithms were compared under identical data conditions.

The accuracy of the SAW tension sensor can be evaluated by the magnitude of the average error in the output, and the formula is as follows:

(23) $$\sigma =\frac{\frac{1}{n}\sum_{i=1}^{n}\left|x_{i}-\bar{x}\right|}{\bar{x}}$$

**Figure 8:**
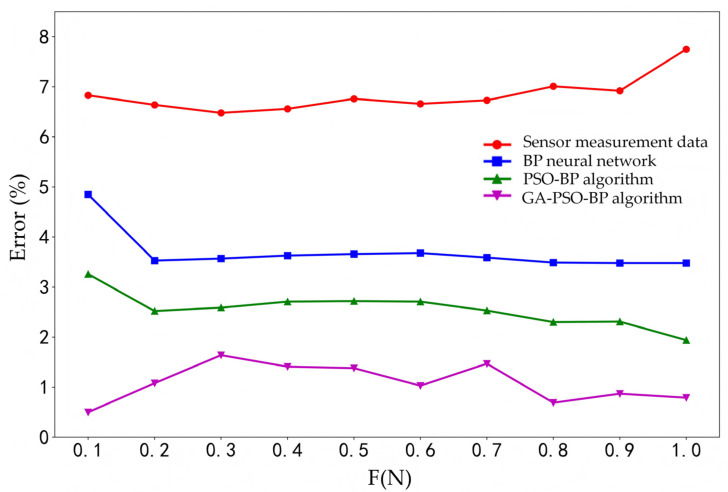
Comparison of the average error of sensor measurement data and three algorithms.

The smaller the value of x is, the less impact this temperature has on the SAW tension sensor, and it also indicates that the accuracy of the SAW tension sensor is higher. As shown in Table 8, the average error of the sensor measurement data is 6.834%, while the average error of the data optimized by the GA-PSO-BP algorithm is 1.086 or even better. And the average error of the GA-PSO-BP algorithm is significantly lower than that of the BP neural network and the PSO-BP algorithm. This difference can be better illustrated in Figure 8. As a result, the GA-PSO-BP algorithm for the temperature compensation of the SAW tension sensor is better.

**Figure 9:**
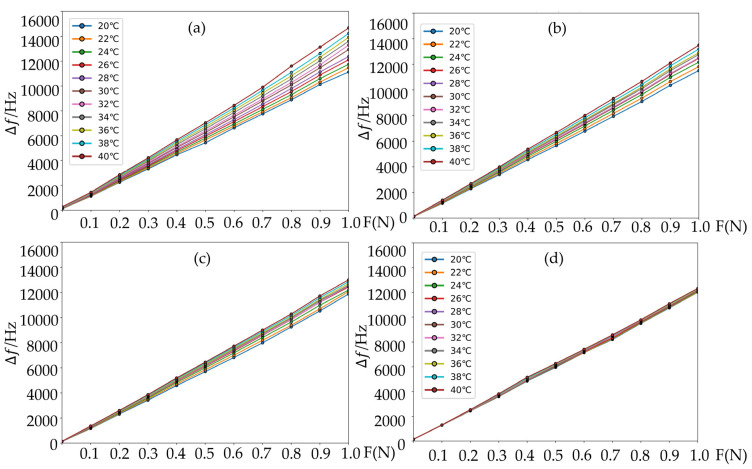
Curve fitting of the temperature-compensated measurement data using different algorithms. (**a**) Original data; (**b**) BP neural network; (**c**) PSO-BP algorithm; (**d**) GA-PSO-BP algorithm.

**Figure 10:**
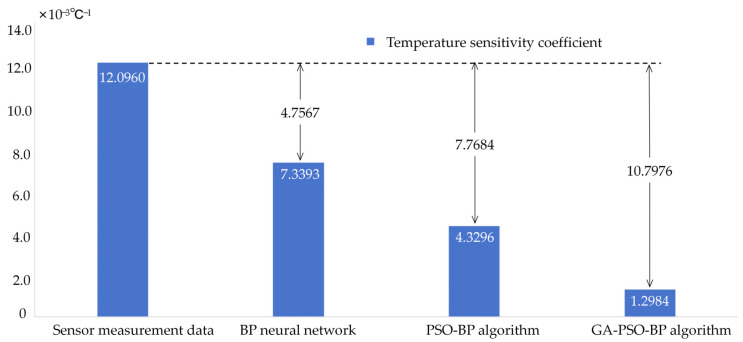
Comparison of temperature sensitivity coefficients of different algorithms.

As shown in Figure 9, the data optimized by the GA-PSO-BP algorithm significantly mitigated the temperature impact on the SAW tension sensor. The temperature sensitivity coefficient was also reduced by an order of magnitude compared to the original data, as indicated in the equation. Figure 10 presents a comparison of the temperature sensitivity coefficients after optimization using different algorithms.

## 5. Conclusions

This paper presents a new continuous tension detection sensor design based on a cantilever beam roller SAW tension sensor to address the demand for silk thread tension control. By optimizing the sensor’s design and structure, the measurement accuracy and stability of silk tension are enhanced. Additionally, a software compensation method is employed to reduce the temperature error of the SAW tension sensor. The conclusions are as follows:

(1) A novel cantilever beam roller SAW tension sensor is developed. The rollers are fixed on the cantilever beam, enabling the silk thread tension to directly act on the cantilever beam, thereby better conducting to the SAW sensor. This structure can significantly enhance the sensitivity of the transmission of silk thread tension to the SAW tension sensor.
(2) A SAW device is created using quartz as the piezoelectric substrate, achieving a target bandwidth of 2.04% at a center frequency of 60 MHz. To suppress parasitic responses of SAW, the sensor design incorporates an unbalanced split-electrode IDT structure, along with appropriate input and output IDTs. To determine the optimal IDT placement, COMSOL-FEM is utilized for modeling and simulation. Based on the simulation results, the significant strain and minimal deformation positions are selected to place the IDTs.
(3) To address the temperature impact on SAW tension sensors, this paper applied the GA-PSO-BP algorithm. This algorithm integrates the global search capability of genetic algorithms (GA) with the local optimization strength of particle swarm optimization (PSO). It effectively reduced the temperature sensitivity coefficient to $1.2984\times 10^{-3}\;{{}^{\circ}C}^{-1}$, marking a significant improvement over the original data and other traditional algorithms. Specifically, compared to traditional BP networks and PSO-BP algorithms, the reductions achieved are $6.0409\times 10^{-3}\;{{}^{\circ}C}^{-1}$ and $3.0312\times 10^{-3}\;{{}^{\circ}C}^{-1}$, respectively. The average output error of the optimized data is reduced by 5.748% compared to the sensor measurement data. The average output error of the optimized data is reduced by 5.748% compared to the sensor measurement data, and it is also lower than both the BP neural network and the PSO-BP algorithm.

## Figures and Tables

**Table 1:** Parameters in BP neural network.

Symbol	Parameter Description
z	weighted input
w	weight
b	biase
a	activation value
$y_{j}$	actual output
$y_{j1}$	predicted output
δ	error
$\sigma^{\prime }\left(z_{j}^{m}\right)$	the derivative of the activation function
η	learning rate

**Table 2:** Parameters in PSO-BP algorithm.

Symbol	Parameter Description
D	dimension
l	number of nodes in input layer of the neural network
m	number of nodes in hidden layer of the neural network
n	number of nodes in output layer of the neural network
ω	inertia weight
$c_{1},c_{2}$	learning factor
$r_{1},r_{2}$	random numbers within the range of 0 to 1

**Table 3:** Relevant parameters to the formulas.

Symbol	Parameter Description
τ	the time length of the input transducer
$H_{in}(f)$	input frequency response of IDT
$H_{out}(f)$	output frequency response of IDT
W	maximum aperture of IDT
$x_{m}$	*m* finger pair of the output overlap
BW	bandwidth
${\;N}_{out}$	output IDT finger pairs
${\;N}_{in}$	input IDT finger pairs
$r_{1},r_{2}$	random numbers within the range of 0 to 1

**Table 4:** Design parameters of the SAW tension sensor.

Center Frequency (MHz)	Band Width (MHz)	Aperture Width of IDT (μm)	Electrode Width (μm)	Spacing Between Electrodes (μm)	Width of Junction Bridge (mm)	Number of Electrodes	
						Input IDT	Output IDT
60	1.2288	3588.7333	a = 3.2896 c = 9.8687	b = 6.5791 d = 6.5791	0.5	59	24

**Table 5:** The materials and design parameters of the SAW tension sensor.

Parameters	Value
Permittivity	3.9
Elastic matrix $(10^{11}\;N/m^{-2}$)	$\left[\begin{array}{cccccc} 0.865 & 0.067 & 0.119 & -0.179 & 0 & 0\\ 0.067 & 0.865 & 0.119 & 0.179 & 0 & 0\\ 0.119 & 0.119 & 1.072 & 0 & 0 & 0\\ -0.179 & 0.179 & 0 & 0.579 & 0 & 0\\ 0 & 0 & 0 & 0 & 0.579 & -0.179\\ 0 & 0 & 0 & 0 & -0.179 & 0.399 \end{array}\right]$
$\mathrm{Densities}\;(g/{cm}^{3})$	2.2
Base length (mm)	30
Base width (mm)	5.0
Base height (mm)	0.5

**Table 6:** Measurement data of the SAW tension sensor.

F(N)	T (°C)										
	20	22	24	26	28	30	32	34	36	38	40
	Δf(Hz)										
0.0	121	134	142	155	169	183	199	213	231	253	276
0.1	1128	1155	1187	1214	1253	1288	1316	1345	1378	1413	1446
0.2	2246	2298	2363	2419	2486	2553	2608	2672	2726	2795	2868
0.3	3325	3403	3499	3575	3658	3762	3837	3941	4027	4114	4217
0.4	4461	4559	4678	4796	4901	5046	5140	5288	5406	5531	5663
0.5	5430	5657	5804	5958	6083	6261	6387	6561	6711	6876	7037
0.6	6629	6774	6942	7135	7294	7514	7653	7869	8056	8241	8440
0.7	7755	7921	8119	8339	8540	8799	8965	9202	9439	9659	9906
0.8	8888	9063	9300	9550	9791	10,082	10,283	10,562	10,818	11,094	11,603
0.9	10,125	10,318	10,598	10,883	11,134	11,466	11,703	12,032	12,306	12,613	13,132
1.0	11,127	11,472	11,748	12,118	12,368	12,934	13,295	13,601	13,918	14,235	14,678

**Table 7:** The compensated data of the SAW tension sensor obtained by the GA-PSO-BP algorithm.

F(N)	T (°C)										
	20	22	24	26	28	30	32	34	36	38	40
	Δf(Hz)										
0.0	128	130	133	135	137	138	139	142	144	145	148
0.1	1275	1281	1285	1287	1289	1291	1293	1295	1296	1299	1300
0.2	2429	2440	2453	2466	2474	2484	2489	2500	2512	2520	2533
0.3	3579	3601	3625	3649	3666	3683	3693	3728	3764	3782	3812
0.4	4843	4883	4905	4926	4944	4962	4970	5016	5033	5082	5152
0.5	5949	5998	6009	6037	6073	6103	6152	6160	6188	6227	6261
0.6	7138	7151	7198	7215	7248	7284	7297	7326	7355	7384	7403
0.7	8198	8249	8293	8331	8443	8450	8459	8491	8530	8539	8572
0.8	9498	9531	9574	9618	9679	9699	9714	9743	9751	9776	9792
0.9	10,742	10,781	10,812	10,854	10,914	10,942	10,955	10,971	11,002	11,057	11,091
1.0	12,003	12,025	12,075	12,144	12,164	12,182	12,233	12,251	12,264	12,307	12,323

**Table 8:** The average error of sensor measurement data and three algorithms.

Error (%)	F (N)										
	0.1	0.2	0.3	0.4	0.5	0.6	0.7	0.8	0.9	1.0	Avg
Sensor measurement data (experiment)	6.83	6.64	6.48	6.56	6.76	6.66	6.73	7.01	6.92	7.75	6.834
BP neural network	4.85	3.53	3.57	3.63	3.66	3.68	3.59	3.49	3.48	3.48	3.696
PSO-BP algorithm	3.26	2.52	2.59	2.71	2.72	2.71	2.53	2.30	2.31	1.94	2.559
GA-PSO-BP algorithm	0.5	1.08	1.64	1.41	1.38	1.03	1.47	0.69	0.87	0.79	1.086

## Data Availability

The original contributions presented in this study are included in the article; further inquiries can be directed to the corresponding authors.
